# Metformin sensitizes the response of oral squamous cell carcinoma to cisplatin treatment through inhibition of NF-κB/HIF-1α signal axis

**DOI:** 10.1038/srep35788

**Published:** 2016-10-20

**Authors:** Xiaofeng Qi, Wengguang Xu, Junqi Xie, Yufeng Wang, Shengwei Han, Zheng Wei, Yanhong Ni, Yingchun Dong, Wei Han

**Affiliations:** 1 Department of Oral and Maxillofacial Surgery, Nanjing Stomatological Hospital, Medical School of Nanjing University, No 30 Zhongyang Road, Nanjing, China; 2 Central Laboratory of Stomatology, Nanjing Stomatological Hospital, Medical School of Nanjing University, Nanjing, P.R China; 3 Department of Anesthesia, Nanjing Stomatological Hospital, Medical School of Nanjing University, Nanjing, P.R China

## Abstract

Resistance towards chemotherapy is a common complication in treatment of oral cancers, which leads to treatment failure and poor outcome. In recent years, a growing body of evidence has shown that tumour hypoxia significantly contributes to chemoresistance. Metformin, a widely used oral hypoglycaemic drug, can reportedly potentiate the efficacy of chemotherapeutic drugs in various cancers; however, the underlying mechanisms are intricate and have not been fully understood. In this study, we explored the role of metformin in chemosensitivity of oral squamous cell carcinoma cells (OSCC) to cisplatin both *in vitro* and *in vivo*, and attempted to elucidate its possible underlying mechanisms. Encouragingly, we found that metformin synergistically enhanced cisplatin cytotoxicity and reversed the chemoresistance to certain extent. This mechanism could likely be related with inhibition of the NF-κB/HIF-1α signal axis and lead to the downregulation of hypoxia-regulated genes products. Therefore, metformin could serve as a chemosensitiser for cisplatin-based regimens for OSCC, thereby providing a theoretical basis for future use in the treatment of oral cancers.

Oral cancer ranks as the 11th most prevalent cancer worldwide, and among all oral malignancies, oral squamous cell carcinoma (OSCC) is the most predominant, constituting about 90% of all oral cancers^1^,^2^,^3^. In spite of rapid advances in surgery and adjuvant therapy, the prognosis for oral cancer remains poor, with the 5-year survival rate maintained at 30–60%^4^,^5^,^6^. According to the protocol of treatment guidelines for oral cancers, chemotherapy would be recommended for patients with loco-regionally advanced diseases especially if aggressive features were found, such as positive surgical margins and extracapsular spread in lymph node metastasis^7^. Currently, cisplatin-based chemotherapeutic regimens have been the most widely and frequently used adjuvant treatments against OSCC^8^; however, chemotherapeutic drugs do not specifically target tumour cells, and thus may have deleterious effects on normal cells. In addition to this, chemotherapy resistance tends to develop as the treatment continues, which is the main cause of tumour relapse and treatment failure^9^. It is therefore crucial to seek effective agents that could be used in combination with present chemotherapeutic regimens to simultaneously reduce the doses and sensitize their therapeutic effect.

To date, accumulating evidence indicates that tumour hypoxia, a pivotal feature of solid tumours on account of defective tumour vasculature, correlates closely with resistance to chemotherapy^10^,^11^. The major transcription factor in response to hypoxic stress is believed to be hypoxia-inducible factor-1 (HIF-1), which is a heterodimer composed of an HIF-1α and a constitutively expressed HIF-1β subunit^12^. Recent studies have validated a close connection between HIF-1α expression and chemoresistance in multiple malignancies^13^,^14^,^15^,^16^. Another characteristic feature of the tumour microenvironment is inflammation, which contributes to carcinogenesis, cancer invasion, and metastasis^17^. An essential element responsible for the regulation of inflammatory response is nuclear factor-kappa B (NF-κB), composed of a family of subunits called RelA/p65, RelB, cRel, p100/p52, and p105/p50^18^. It is evident that a crosstalk does exist between these two key molecular players involved in hypoxia and inflammation. In our previous study, we proved that NF-κB was located upstream of the HIF-1α promoter^19^, with the function of regulating HIF-1α transcription, which was fairly consistent with some of the previous studies^18^,^20^,^21^. The crucial role of HIF-1α in chemoresistance indicates that an effective approach could be provided to overcome chemoresistance through the inhibition of the upstream key factor—NF-κB.

Metformin (1,1-dimethylbiguanide hydrochloride), the first-line therapeutic drug for type 2 diabetes mellitus in clinical practice, is a biguanide derived from the French lilac *Galega officinalis*. Recently, a series of epidemiologic studies suggested that metformin may reduce cancer risk and improve outcomes of many types of cancers^22^,^23^,^24^. Moreover, encouraging outcomes from a few clinical studies have shown that metformin may contribute to a better treatment response among cancer patients^25^,^26^. Besides, several studies have shown that metformin somehow exerts synergistic effects on the efficacy of chemotherapy and lowers the dose of therapeutic drugs^27^,^28^, which implies that metformin could be recommended as a neoadjuvant anticancer agent combined with traditional chemotherapeutic drugs. In this study, we proved that metformin could sensitize the response of OSCC to cisplatin treatment through inhibition of NF-κB/HIF-1α signaling both *in vitro* and *in vivo*, which might serve as a novel chemo-regimen for tumour microenvironment-targeted therapy.

## Results

### Hypoxia potentiates chemoresistance to cisplatin in OSCC cells

**Figure 1:**
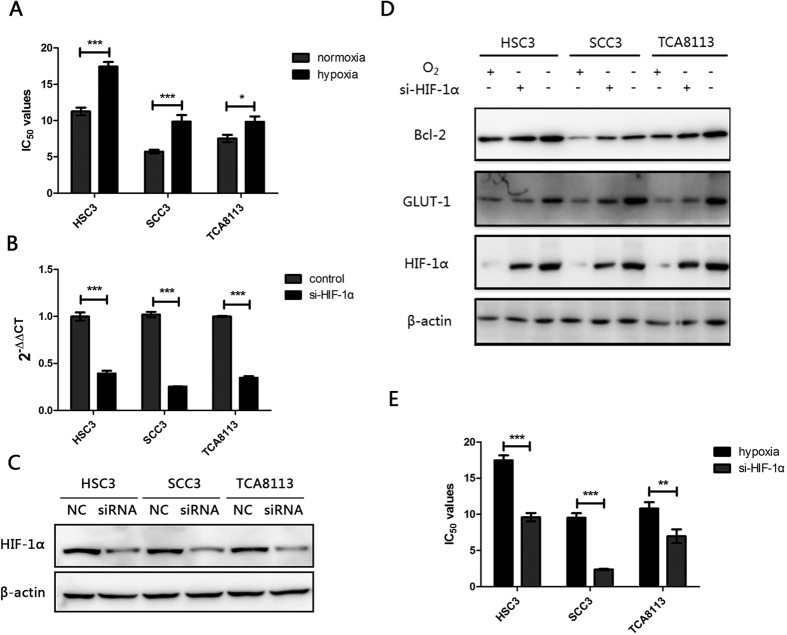
Hypoxia potentiates chemoresistance to cisplatin in OSCC cells. (**A**) HSC3, SCC3, and TCA8113 cells were treated with cisplatin under normoxic (20% O_2_) or hypoxic (1% O_2_) conditions for 48 h. The 50% inhibitory concentration (IC_50_) values of cells was analyzed by MTT assay. Absorbance was measured at 570 nm (A570). (**B**) Expression of HIF-1α mRNA in HSC3, SCC3, and TCA8113 cells transfected with or without HIF-1α siRNA under hypoxic conditions for 48 h. (**C**) Expression of HIF-1α protein in HSC3, SCC3, and TCA8113 cells transfected with or without HIF-1α siRNA under hypoxic conditions for 48 h. Full-length blots are presented in Supplementary Figure S1. (**D**) Expression of Bcl-2, GLUT-1, and HIF-1α in HSC3, SCC3, and TCA8113 cells transfected with or without HIF-1α siRNA under hypoxic conditions for 48 h. Full-length blots are presented in Supplementary Figure S2. (**E**) The IC_50_ values of cells transfected with or without HIF-1α siRNA were analysed by MTT assay under hypoxia. All experiments were repeated at least three times (*p ≤ 0.05; **p ≤ 0.01; ***p ≤ 0.001).

Hypoxia has been shown to lead to chemoresistance in many representative cell lines as reported in previous studies^13^,^14^,^15^,^16^. We initially confirmed whether hypoxia had the capacity to decrease the sensitivity of OSCC cells to cisplatin. The effects of cisplatin on the growth of OSCC cells under normoxic (20% O_2_) or hypoxic (1% O_2_) conditions were evaluated by determining the cell viability via an MTT assay. The results showed that the 50% inhibitory concentration (IC_50_) values of HSC3, SCC3, and TCA8113 cells exposed to hypoxia were significantly higher than those exposed to normoxia following a 48-h cisplatin treatment (Fig. 1A and Table 1). HIF-1α is the key regulator of cellular adaptation to hypoxia. To further investigate whether HIF-1α plays a critical role in chemoresistance induced by hypoxia, *HIF-1α* was knocked down by using a small interfering RNA (siRNA) and further treated with cisplatin. Both mRNA and protein levels of HIF-1α in HSC3, SCC3, and TCA8113 cells were notably decreased after transfection with si-HIF-1α (Fig. 1B,C, Supplementary Figure S1). Moreover, a remarkable decrease was observed in the expression levels of two main downstream genes—glucose transporter 1 (*GLUT1*) and B cell lymphoma-2 (*Bcl-2*) (Fig. 1D, Supplementary Figure S2). Subsequently, the results of MTT assay showed that the values of IC_50_ obtained in the si-HIF-1α groups were observably decreased than those in control groups under hypoxia following a 48-h cisplatin treatment (Table 2 and Fig. 1E). Taken together, these results indicate that hypoxia might induce chemoresistance of OSCC cells to the treatment of cisplatin, and key transcriptional factor HIF-1α plays a prominent role during this process.

### Metformin downregulates HIF-1α expression and inhibits *HIF-1* transcriptional activity in hypoxia

**Figure 2:**
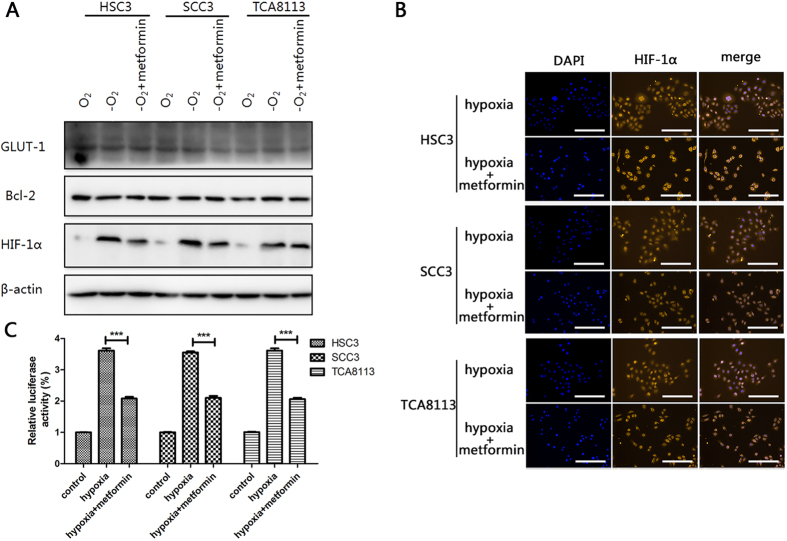
Metformin downregulates HIF-1α expression and inhibits HIF-1 transcriptional activity under hypoxic conditions. (**A**) Expression of GLUT-1, Bcl-2, and HIF-1α in HSC3, SCC3, and TCA8113 cells treated with or without metformin (10 μM) under hypoxic or normoxic conditions for 48 h. Full-length blots are presented in Supplementary Figure S3. (**B**) Representative images of confocal microscopy for HIF-1α expression in HSC3, SCC3, and TCA8113 cells treated with metformin (10 μM). (**C**) HSC3, SCC3, and TCA8113 cells were transfected with PGL6-3 × HRE and cultured under hypoxic conditions, and treated with metformin 48 h after transfection. Relative luciferase activity was assayed subsequently. Values are presented as means ± SD from three independent experiments. (*p ≤ 0.05; **p ≤ 0.01; ***p ≤ 0.001).

To explore the effect of metformin on the regulation of HIF-1α expression, HSC3, SCC3, and TCA8113 cells were all treated with metformin under hypoxic conditions. As shown in Fig. 2A and Supplementary Figure S3, HIF-1α protein accumulation was notably inhibited by metformin treatment in hypoxic conditions. Moreover, the protein expressions of both *GLUT1* and *Bcl-2* (target genes of *HIF-1*) were also reduced, indicating a decrease in transcriptional activity of *HIF-1*. Accounting for the basic low levels of HIF-1α under normoxic conditions, most of the following experiments were performed under hypoxic conditions. To further determine whether the reduction of HIF-1α protein expression could lead to a decrease in *HIF-1* transcriptional activity, immunofluorescence assays were conducted. Our results proved that hypoxia-induced HIF-1α protein accumulations and nuclear translocation dramatically decreased with metformin treatment (Fig. 2B). Finally, to elucidate the role of metformin in HIF-1 regulation, we inserted a sequence counting three copies of HIF-1-binding hypoxia response element (HRE) into a PGL6 plasmid (PGL6-3 × HRE). After OSCC cells were transfected with PGL6-3 × HRE and cultured under hypoxic conditions along with metformin treatment, we observed that metformin dramatically inhibited hypoxia-induced luciferase gene expression (Fig. 2C), which indicated that metformin could exert its effect on luciferase gene expression in an HIF-1-dependent manner. Collectively, these data suggest that metformin inhibits *HIF-1* transcriptional activity by suppressing HIF-1α in OSCC cells under hypoxic conditions.

### Metformin inhibits the activation of NF-κB under hypoxic conditions

**Figure 3:**
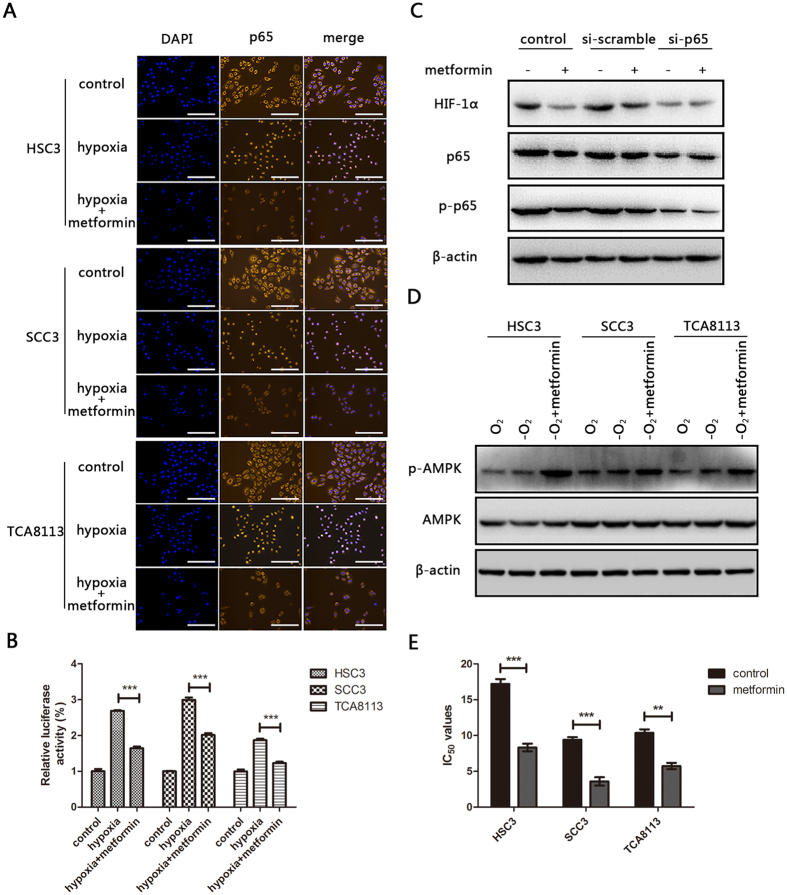
Metformin inhibits the activation of NF-κB under hypoxic conditions. (**A**) Representative images of confocal microscopy for p65 expression in HSC3, SCC3, and TCA8113 cells treated with metformin (10 μM) under normoxic or hypoxic conditions for 24 h. (**B**) HSC3, SCC3, and TCA8113 cells were transfected with pNF-κB-Luc plasmid and cultured under hypoxic conditions treated with or without metformin 48 h after transfection. Relative luciferase activity was assessed subsequently. (**C**) Expression of HIF-1α, p65, and p-p65 in HSC3 cells transfected with or without p65 siRNA under hypoxic conditions for 48 h. Full-length blots are presented in Supplementary Figure S4. (**D**) Expression of p-AMPK and AMPK in HSC3, SCC3, and TCA8113 cells was detected under normoxic conditions, hypoxic conditions treated with or without metformin for 48 h. Full-length blots are presented in Supplementary Figure S5. (**E**) The IC_50_ values of cells treated with or without metformin (10 μM) under hypoxia for 48 h was analysed by MTT assay. All experiments were repeated at least three times (*p ≤ 0.05; **p ≤ 0.01; ***p ≤ 0.001).

NF-κB is a direct modulator of HIF-1α expression, which has been validated in previous reports^19^,^20^,^21^. Upon bioinformatics-based analysis, a potential transcription factor-NF-κB binding site was identified in the HIF-1α promoter. Immunofluorescence assays showed that metformin actually influenced the activation of NF-κB. As results of immunofluorescence shown in Fig. 3A, p65, a major functional subunit of NF-κB was predominantly located in the cytoplasm under normoxic conditions. In contrast, it was translocated to the nucleus where it accumulated, under hypoxic conditions. Treatment of cells with metformin prevented the hypoxia-induced translocation and accumulation of p65 in the nucleus. Moreover, the results of luciferase assay proved that metformin significantly inhibited NF-κB mediated luciferase gene expression (Fig. 3B). To further determine whether metformin induced HIF-1α inhibition via the NF-κB signal, OSCCs were transfected with si-p65 or si-scramble, respectively, and relevant proteins’ expressions were evaluated by western blotting. Metformin treatment resulted in reduced expression of phosphorylated p65 (p-p65) and HIF-1α in both control and si-scramble groups, whereas no significant changes were detected in the si-p65 treated group (Fig. 3C, Supplementary Figure S4). Activation of AMPK was reported to have the effect on inhibiting NF-κB signal previously^29^, thus it was interesting to investigate whether metformin treatment on OSCC could also regulate AMPK signal. Encouragingly, under hypoxia condition, metformin strongly increased the expression of phosphorylated AMPK, which might partially explain why it could have the effect on inhibiting NF-κB signal (Fig. 3D, Supplementary Figure S5). Together, these results indicate that metformin has the potential to inhibit NF-κB activation and subsequently suppress the expression of HIF-1α in OSCC cells under hypoxic conditions.

### Metformin sensitizes OSCC cells to cisplatin under hypoxic conditions

**Figure 4:**
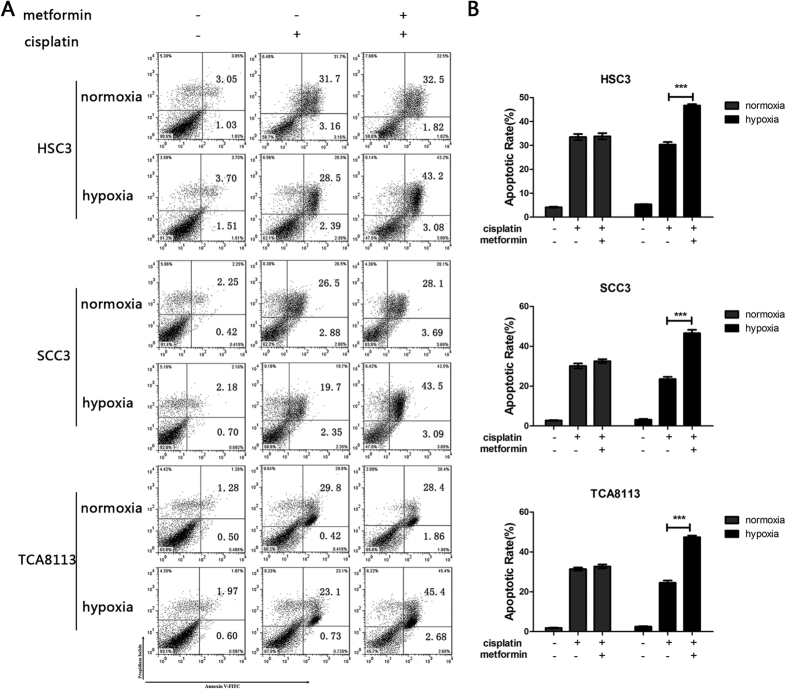
Metformin sensitizes OSCC cells to cisplatin under hypoxic conditions. (**A**) HSC3, SCC3, and TCA8113 cells were treated with metformin (10 μM) and/or cisplatin (15 μM) for 48 h, and apoptotic cells were detected by Annexin V-FITC and PI staining. (**B**) Apoptotic rate of cells was represented according to results of (**A**). Values are presented as means ± SD from three independent experiments. *p* < 0.001 compared to HSC3, SCC3, and TCA8113 cells under hypoxic conditions treated with cisplatin alone.

On the basis of the above results, it was clear that metformin could inhibit the NF-κB/HIF-1α signal axis in OSCC cells under hypoxia. Therefore, we investigated whether the drug could sensitize OSCC cells to cisplatin treatment under hypoxic conditions. OSCC cells were exposed to cisplatin alone or in combination with metformin under normoxic or hypoxic conditions respectively. MTT cell viability assay showed that the IC_50_ values obtained in metformin co-treated groups were significantly lower than the control group, under hypoxic conditions (Table 3, Fig. 3E). Cell apoptosis was detected 48 h later by Annexin V-FITC and PI double staining (Fig. 4A). The addition of metformin significantly enhanced cell apoptosis under hypoxic conditions. However, no markedly increased cell apoptotic rate was found in cells co-treated with cisplatin and metformin under normoxic conditions (Fig. 4B). In general, these results revealed that metformin was equipped to increase the chemo-sensitivity of OSCC cells to cisplatin.

### Metformin enhanced the chemotherapy efficacy of cisplatin *in vivo*

**Figure 5:**
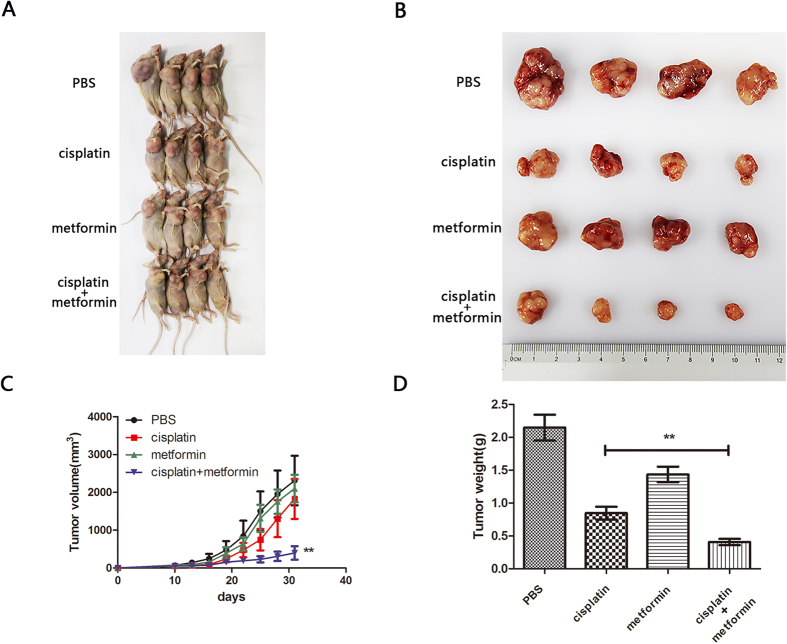
Metformin enhanced the chemotherapeutic efficacy of cisplatin *in vivo*. (**A**) The images of HSC3 xenograft mice treated with PBS control, cisplatin (10 mg/kg), metformin (10 mg/kg), and cisplatin + metformin for 21 days, ten days after tumourigenesis are represented. (**B**) The tumour images of HSC3 xenograft mice mentioned in (**A**) were evaluated. (**C**) The growth curves of PBS control, cisplatin (10 mg/kg), metformin (10 mg/kg), and cisplatin + metformin treated HSC3 xenograft tumours. (**D**) The tumour weight of each group was analysed. (*p ≤ 0.05; **p ≤ 0.01; ***p ≤ 0.001).

To identify whether metformin enhanced the chemotherapy efficacy *in vivo*, an OSCC subcutaneous xenograft model was established in nude mice. As shown in Fig. 5A,B, compared with the control group, a significant reduction in tumour volume was observed in both the cisplatin alone and cisplatin plus metformin groups. More importantly, cisplatin plus metformin exhibited a more powerful anticancer effect than cisplatin alone in view of the significantly smaller tumour volume in this group.

**Figure 6:**
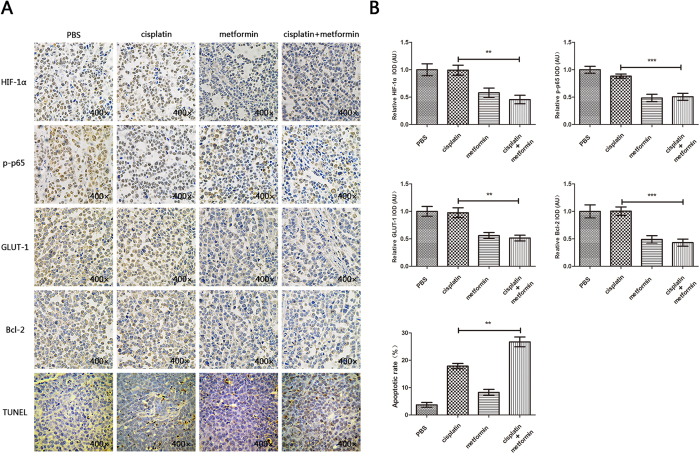
Metformin inhibited the activation of NF-κB/HIF-1α signal *in vivo*. (**A**) IHC staining showed different expressions of HIF-1α, p-p65, GLUT1, and Bcl-2 in the cisplatin-treated, metformin-treated, and cisplatin + metformin-treated groups. TUNEL assay showed variable apoptotic conditions in each group. (**B**) Statistical analysis showed significantly lower expression of NF-κB/HIF-1α signal protein change in metformin and metformin plus cisplatin treated groups. (*p ≤ 0.05; **p ≤ 0.01; ***p ≤ 0.001).

Next, we examined the expression levels of NF-κB/HIF-1α axis-related proteins (p65, p-p65, HIF-1α, GLUT1, Bcl-2) by immunohistochemistry in animal model samples. As expected, the expression levels of p-p65, HIF-1α, GLUT1, Bcl-2 were remarkably reduced in the combination treatment group, suggesting the importance of NF-κB/HIF-1α signal-axis inhibition to sensitize cisplatin treatment. Results of the TUNEL assay further confirmed more TUNEL-positive cells in the cisplatin plus metformin group, indicating a stronger pro-apoptotic and synergistic effect (Fig. 6). Collectively, these results suggested that metformin treatment to OSCC could produce a synergistic effect on the efficacy of cisplatin via inhibition of the NF-κB/HIF-1α signal axis, which might provide a novel chemotherapeutic regimen for OSCC.

## Discussion

Despite surgery, chemotherapy and radiation are widely regarded as the most effective modalities to treat OSCC, unfortunately, a prominent number of OSCC cases are reported to be resistant or partially resistant to first-line cisplatin-based chemotherapeutic regimens for unknown reasons^30^. Thus, there is an urgent need to seek alternative potential drugs to enhance the efficacy of cisplatin-based treatments in OSCC. Our study showed that metformin, a first-line regimen of type 2 diabetes mellitus, had the potential to synergize with cisplatin to induce higher pro-apoptotic and anti-proliferative activity, through inhibition of NF-κB/HIF-1α axis in OSCC cells.

Tumour hypoxia, a pivotal characteristic of the neoplastic microenvironment, has been known to negatively affect efficacy of therapy^31^. As a key regulator of cellular adaptation to hypoxia, hypoxia-inducible factors (HIFs) play an important role in the progression of various cancers^32^. In this study, we initially validated that hypoxia and HIF-1α decreased sensitivity of cancer cells to cisplatin in OSCC cell lines. The IC_50_ values of cisplatin under hypoxic conditions were much higher than those under normoxic conditions. More importantly, HIF-1α knockdown could significantly decrease the IC_50_ of cisplatin under hypoxic conditions. Thus, circumventing or reversing the hypoxia-induced chemoresistance in OSCC cells is tricky and complex.

Metformin is a widely used and well-tolerated drug for type 2 diabetes, which has garnered much attention in the field of cancer therapy in recent years. Several preclinical studies have attempted to identify mechanisms of metformin’s anticancer effects. Interestingly, accumulating studies indicate that metformin could not only exert anticancer effects alone but also enhance response to chemotherapy synergistically; however, the underlying mechanisms are complicated and have not been fully understood^33^. To the best of our knowledge, our study is the first report in the field of OSCC to elucidate synergy between metformin and cisplatin both *in vitro* and *in vivo*. By adding metformin to the chemotherapeutic regimen, both HIF-1α expression and HIF-1 transcriptional activity was downregulated, followed by lower expressions of GLUT1 and Bcl-2. GLUT1 is an applicable marker of hypoxia and glucose metabolism, which has been found over-expressed in a variety of cancers^34^. The downregulation of GLUT1 in our study has indicated that metformin plus cisplatin can negatively affect the energy supply for cell viability, by reducing glucose transport across cellular membranes, and thereby inducing cellular apoptosis. Some studies also suggested that higher expression of Bcl-2 resulted in resistance against chemotherapy^35^,^36^. In the present study, the Bcl-2 expression was inhibited, suggesting that metformin use reversed the chemoresistance to a certain extent.

In fact, a number of studies have shown that the efficacy of therapeutic drugs could be potentiated by metformin; some studies have even attempted to elucidate the underlying mechanisms. In accordance with our results, Kim *et al*. revealed that metformin could activate AMPK and suppresses MDR1 expression in MCF-7/adr cells by inhibiting the activation of NF-κB and CREB^37^. Li *et al*. found that metformin potentiates the effects of tyrosine kinase inhibitors (TKIs) in patients with non–small cell lung cancer through inhibition of IL-6 signalling^38^,^39^. Alternatively, Hanna *et al*. reported that metformin synergistically enhanced anticancer effect of paclitaxel in endometrial cancer cells through inhibition of the mTOR pathway^27^. Interestingly, however, some studies have drawn slightly different conclusions from ours, in that, metformin may exhibit a cyto-protective effect against cisplatin. Damelin *et al*. demonstrated that metformin could lead to low cisplatin-DNA complex formation, thus exerting an antagonistic effect against cisplatin cytotoxicity in oesophageal SCC cells^40^. In fact, the seemingly contradictory conclusions are understandable, because in their studies, the *in-vitro* toxicity detection is conducted under conditions of ambient air in common cell culture medium with abundant nutrition. Yu *et al*. showed that under glucose-deprivation conditions, metformin enhanced cisplatin cytotoxicity in an oesophageal SCC cell line, which could be comparable to our results^41^.

We believe that a combination strategy of metformin with conventional chemotherapy may be more robust than metformin as monotherapy. In the present study, metformin by itself did not exhibit a marked anticancer effect *in vitro* or *in vivo*, perhaps because of the relative low concentration of metformin administered in our study. Researchers in clinical translational medicine have found that a high dose of metformin is needed to impede tumour biology, although it presently remains inconclusive whether such a high dose of metformin would have any adverse effects^42^,^43^. Another likely explanation for this phenomenon is metformin’s complicated and tumour-specific anticancer effect. Consistent with our study, some researchers investigated the effect of metformin on colorectal cancer cells: metformin did not elicit pro-apoptotic and anti-proliferative effects *in vitro* and *in vivo*^44^. Additionally, two clinical trials on metastatic pancreatic cancer showed no favourable outcomes with metformin use^45^,^46^. Therefore, metformin use may not be a feasible choice for all cancer types.

In summary, to our best knowledge, our study reported for the first time that metformin could potentially induce chemosensitivity of OSCC cells to cisplatin through the inhibition of NF-κB/HIF-1α axis and HIF-1-regulated gene products. Thus, a combination strategy of metformin with cisplatin-based chemotherapy will shed light on the improvement of chemotherapeutic efficacy for future OSCC treatment.

## Materials and Methods

### Ethics statement

All animal experiments complied with national and international guidelines. Institutional review board approval was obtained from Nanjing Stomatological Hospital Ethics Committee [approval number, 2015NL-013(KS)].

### Cell line and cell culture

Oral squamous cell carcinoma lines, HSC3, SCC3, and TCA8113, were kindly provided by the Ninth Hospital of Shanghai. They were maintained in Dulbecco’s Modified Eagle’s Medium (DMEM) with 10% fetal bovine serum (FBS) at 37 °C in a humidified atmosphere containing 5% CO_2_. 20% and 1% oxygen were used induce normoxia- or hypoxia-mimicking conditions.

### Reagents and antibodies

Metformin (1,1-dimethylbiguanide hydrochloride), cisplatin 3-(4,5-dimethylthiazol-2-yl)-2, and 5-diphenyltetrazolium bromide solution (MTT) were obtained from Sigma Chemical Inc. (St. Louis, Missouri, USA). Mouse monoclonal anti-human HIF-1α antibody and BCL-2, GLUT-1, p65, p-p65 (phospho S536), AMPK, phosphor-AMPK were obtained from Abcam Inc. (Cambridge, Massachusetts, USA). PCR primers and pGL-HRE Promoter plasmid were synthesised from Invitrogen Corp. (Carlsbad, CA, USA). Annexin V-FITC Apoptosis Detection Kit was procured from KaiJi Inc. (Nanjing, China). DMEM and FBS were purchased from Gibco (Grand Island, NY, USA).

### MTT assay

Cells were incubated in 96-well tissue-culture plates with the indicated reagents for the indicated times. Twenty microliters of 5 mg/mL MTT solution in phosphate-buffered saline (PBS) was then added to each well and incubated for 5 h. The unreactive supernatant was removed from each well after centrifugation and replaced with 200-μl dimethylsulfoxide, after which the plates were again incubated for 10 min in the dark. The absorbance at 570 nm (A570) was measured using a scanning multi-well spectrophotometer (Gene, USA).

### Western blotting analysis

Cells cultured under hypoxic or normoxic conditions were harvested and lysed for 20 min using modified RIPA buffer (5 mM EDTA, 2 mM Na3VO4, 5 mM NaF, 1 mM phenylmethylsulfonyl fluoride) supplemented with a protease inhibitor cocktail (Sigma-Aldrich). Protein extracts were loaded onto a 10% sodium dodecyl sulfate–polyacrylamide gel, electrophoresed, and transferred to a polyvinylidene difluoride membrane. Protein bands were probed with anti-HIF-1α and other experimental primary antibodies at 4 °C overnight after blocking with 5% separated milk, followed by alkaline phosphatase-linked secondary antibody (Cell Signaling Technology, USA) incubation for 1 h at 37 °C. Specific signals were visualized using nitroblue tetrazolium/5-bromo-4-chloro-3- indolylphosphate/buffer (1:1:50).

### Plasmid transfections

The firefly luciferase reporter plasmid PGL6-3 × HRE containing hypoxia-response elements (HREs) was purchased from Promega (Madison, WI, USA). pNF-κB-Luc plasmid was purchased from youbio (Hu nan, China). Cells were transfected using Lipofectamine 2000 transfection reagent (Invitrogen), according to the manufacturer’s protocol. Transfected cells were replated and replenished with vehicle- or reagent-containing medium. Luciferase activity was measured, as previously described^47^.

### Luciferase assay

HSC3, SCC3, and TCA8113 cells were transfected with PGL6-3 × HRE luciferase plasmid, pNF-κB-Luc plasmid or control vectors by using Lipofectamine 2000. A Renilla luciferase plasmid (pRL-CMV from Promega) was co-transfected as an internal control. Cells were collected 48 h after transfection, and the luciferase activities of the cell lysates were measured by using the Dual-luciferase Reporter Assay System (Promega). The data are presented as the mean and standard deviation (SD) of three experimental replicates.

### Immunofluorescence

Cells were plated on glass coverslips into 24-well plates and allowed to attach overnight. The following day, cells were treated with the indicated drugs for the specified times. Cells were fixed with ethanol precooled to 20 °C for 15 min. Coverslips were blocked in 5% bovine serum albumin for 1 h at 37 °C and processed for immunofluorescence with primary anti-HIF-1α and anti-p65 antibodies. Cy3 goat anti-mouse antibody was used as the secondary antibody. DAPI (Life technology, USA; diluted 1:100 in PBS) was used for nuclear staining. Coverslips were then mounted onto glass slides and examined using an inverted fluorescence microscope (Olympus).

### HIF-1α knockdown with siRNA

The siRNAs used to knock down HIF-1α expression in OSCC cells were obtained from GenePharma (Shang Hai, China). We designed three sequences for each target. Cells transfected with different concentrations of each siRNA were then cultured for 48 h. The relative mRNA expression of the target gene was measured by Quantitative real-time PCR. The siRNAs that achieved the most effective knockdown of the targets were selected for further use. The sequences of each siRNA used are listed in Table 4. The siRNAs and non-silencing controls were transfected into cells using Lipofectamine 2000. Transfected cells in fresh media were incubated under the appropriate experimental conditions and harvested for sample preparation.

### Quantitative reverse transcription polymerase chain reaction (qRT-PCR) analysis

Total RNA was isolated using Trizol reagent (Invitrogen), following the manufacturer’s instructions. cDNA was synthesized from the isolated RNA by RT and individually PCR amplified. The primers for HIF-1α were as described previously^48^, and the primers used in the experiments are listed in Table 5. DNA products were separated on 1.5% agarose gels and visualized by ethidium bromide staining under UV light.

### Flow cytometric analysis

Cell apoptosis was detected with the Annexin V-FITC Apoptosis Detection Kit, according to the manufacturer’s instructions. In brief, 2 × 10^5^ cells were collected, washed twice with PBS, resuspended in 500 μl binding buffer, and incubated with 5 μl Annexin V-FITC and 5 μl propidium iodide (PI) for 10 min before analysis on an FACS Calibur flow cytometer (BD Biosciences, USA).

### Nude mice and tumour inoculations

All animal experimental protocols were approved by the Ethics Committee of Nanjing Stomatological Hospital. Mice were maintained under specific pathogen-free conditions (n = 16, 4 mice/group). Immunoincompetent nude mice were injected subcutaneously in the right flank with 100 μL HSC3 (1 × 10^7^/mL; 4 mice/group). The tumour volume was measured every 3 days with a caliper by using the formula (volume = long diameter × short diameter^2^/2). After 10 days, the tumours were injected every 3 days with PBS, cisplatin (10 mg/kg, intraperitoneal injection), metformin (10 mg/kg, intraperitoneal injection), or cisplatin plus metformin. After 3 weeks, the mice were sacrificed and the xenograft tumours were removed for formalin fixation and preparation of paraffin-embedded sections.

### TUNEL assay

Apoptosis (programmed cell death) in the tumour specimens from mice was examined by the TUNEL method using an *in situ* cell death kit (Roche, USA) according to the manufacturer’s protocol. Nuclei were counterstained with DAPI reagent. Positive cells were visualized by laser scanning confocal microscopy (FV-1000, Olympus, Japan).

### Immunohistochemistry

Xenograft tumours were fixed with 10% formalin, embedded in paraffin, sectioned (5-μm thickness), dewaxed with xylene, and then dehydrated in a graded series of ethanol. Specimens of each group were used for stating with HIF-1α (1:200), Bcl-2 (1:300), GLUT-1 (1:300) and p-p65 (phospho S536; 1:200) antibodies. Sections were counterstained with Mayer’s hematoxylin, dehydrated through a graded ethanol series into xylene, and then mounted.

### Statistical analysis

All experiments were performed at least in triplicate. SPSS software (SPSS Inc., Chicago, IL, USA) was used for all statistical analysis. P values less than 0.05 were considered statistically significant. GraphPad Prism 5.0 software package (version 5.01 for Windows, GraphPad Software, Inc., San Diego CA, USA) was used for diagram analysis.

## Additional Information

**How to cite this article**: Qi, X. *et al*. Metformin sensitizes the response of oral squamous cell carcinoma to cisplatin treatment through inhibition of NF-κB/HIF-1α signal axis. *Sci. Rep.* **6**, 35788; doi: 10.1038/srep35788 (2016).

## Supplementary Material

Supplementary Information

## Figures and Tables

**Table 1:** IC_50_ values obtained from dose–response curves to cisplatin in HSC3, SCC3 and TCA8113 cells following 48-h treatment with cisplatin (0–90 uM) and incubation under normoxia or hypoxia.

	Control(normoxia)	pO_2_1%
HSC3	11.26 ± 0.91	17.44 ± 1.10
SCC3	5.73 ± 0.42	9.86 ± 1.55
TCA8113	7.54 ± 0.88	9.83 ± 1.30

(Means ± s.e.m. of 3 independent experiments).

**Table 2:** IC_50_ values obtained from dose–response curves to cisplatin in HSC3, SCC3 and TCA8113 cells following 48-h treatment with cisplatin (0–90 uM) and incubation in hypoxia or hypoxia with the treatment of si-HIF-1α.

	Control(pO_2_1%)	si-HIF-1α(pO_2_1%)
HSC3	17.48 ± 1.17	9.60 ± 1.00
SCC3	9.52 ± 1.11	2.37 ± 0.16
TCA8113	10.82 ± 1.51	6.97 ± 1.65

(Means ± s.e.m. of 3 independent experiments).

**Table 3:** IC5_0_ values obtained from dose–response curves to cisplatin in HSC3, SCC3 and TCA8113 cells following 48-h treatment with cisplatin (0–90 uM) and incubation in hypoxia or hypoxia with the treatment of metformin (10 uM).

	Control(pO_2_1%)	metformin(pO_2_1%)
HSC3	17.21 ± 1.12	8.32 ± 0.92
SCC3	9.41 ± 0.62	3.59 ± 1.02
TCA8113	10.36 ± 0.87	5.73 ± 0.77

(Means ± s.e.m. of 3 independent experiments).

**Table 4:** Primers of Q-PCR GENE Forward Reverse.

HIF-1α	TTTGCTGAAGACACAGAAGCAAAGA	TTGAGGACTTGCGCTTTCAGG
β-actin	CTGGGACGACATGGAGAAAA	AAGGAAGGCTGGAAGAGTGC

**Table 5:** Sequences of siRNA.

GENE	Target site	Sense (5′–3′)	Anti-sense (5′–3′)
HIF-1α	1113	GGCCGCUCAAUUUAUGAAUTT	AUUCAUAAAUUGAGCGGCCTT
	2155	CCACCACUGAUGAAUUAAATT	UUUAAUUCAUCAGUGGUGGTT
	1509	GCUGGAGACACAAUCAUAUTT	AUAUGAUUGUGUCUCCAGCTT
p65	755	GGGAUGAGAUCUUCCUACUTT	AGUAGGAAGAUCUCAUCCCTT
	1455	GCUGCAGUUUGAUGAUGAATT	UUCAUCAUCAAACUGCAGCTT
	1707	CCUCCUUUCAGGAGAUGAATT	UUCAUCUCCUGAAAGGAGGTT
Negative control		UUCUCCGAACGUGUCACGUdTdT	ACGUGACACGUUCGGAGAAdTdT
