# Erratum: Metformin sensitizes the response of oral squamous cell carcinoma to cisplatin treatment through inhibition of NF-κB/HIF-1α signal axis

**DOI:** 10.1038/srep42934

**Published:** 2017-02-24

**Authors:** Xiaofeng Qi, Wenguang Xu, Junqi Xie, Yufeng Wang, Shengwei Han, Zheng Wei, Yanhong Ni, Yingchun Dong, Wei Han

Scientific Reports 6: Article number: 3578810.1038/srep35788; published online: 10 20 2016; updated: 02 24 2017

The original version of this Article contained a typographical error in the spelling of the author Wenguang Xu, which was incorrectly given as Wengguang Xu. This has now been corrected in the PDF and HTML versions of the Article.

