# Supplementary material for: Metformin sensitizes the response of oral squamous cell carcinoma to cisplatin treatment through inhibition of NF-κB/HIF-1α signal axis
Source: Sci Rep. 2016 Oct 20;6:35788. doi: 10.1038/srep35788 (PMC5071902; doi:10.1038/srep35788)

Metformin sensitizes the response of oral squamous cell carcinoma to cisplatin treatment through inhibition of NF-κB/HIF-1α signal axis

Qi Xiaofeng[1], Xu Wenguang[1], Xie Junqi[1], Wang Yufeng[1], Han Shengwei[1], Wei Zheng[1], Ni Yanhong[2], Dong Yingchun[3], Han Wei[1,2*]

1 Department of Oral and Maxillofacial Surgery, Nanjing Stomatological Hospital, Medical School of Nanjing University, No 30 Zhongyang Road, Nanjing, China

2 Central Laboratory of Stomatology, Nanjing Stomatological Hospital, Medical School of Nanjing University, Nanjing, P.R China

3 Department of Anesthesia, Nanjing Stomatological Hospital, Medical School of Nanjing University, Nanjing, P.R China

The first two authors contribute equally to this paper.

Correspondence to:

Han Wei

Email: doctorhanwei@hotmail.com

Tel: +86 25 83620140

Fax: +86 25 83620173

Supplementary File

Supplementary Figure S1. Expression of HIF-1α protein in HSC3, SCC3, TCA8113 and CAL27 (The band of this cell line was not displayed in the cropped blots of the main paper) cells transfected with or without HIF-1α siRNA under hypoxic conditions for 48 h.

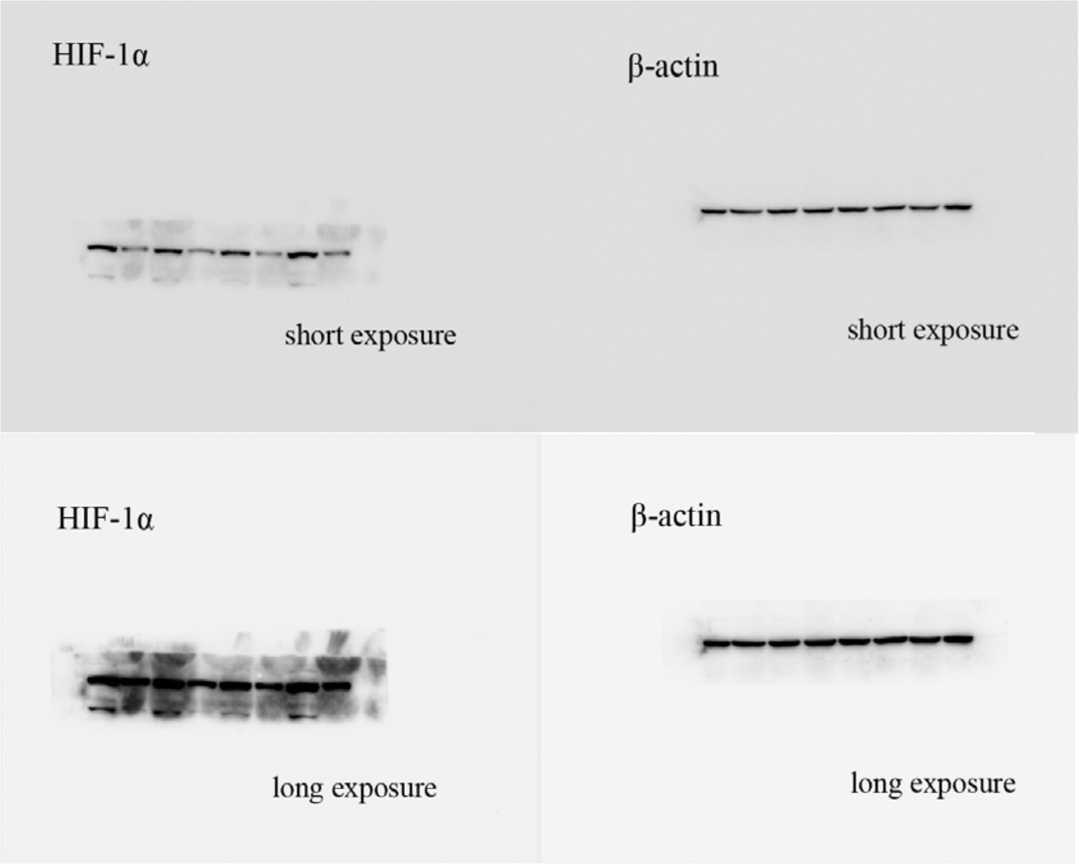

Supplementary Figure S2. Expression of Bcl-2, GLUT-1, and HIF-1α in HSC3, SCC3, TCA8113 and CAL27 (The band of this cell line was not displayed in the cropped blots of the main paper) cells transfected with or without HIF-1α siRNA under hypoxic conditions for 48 h.

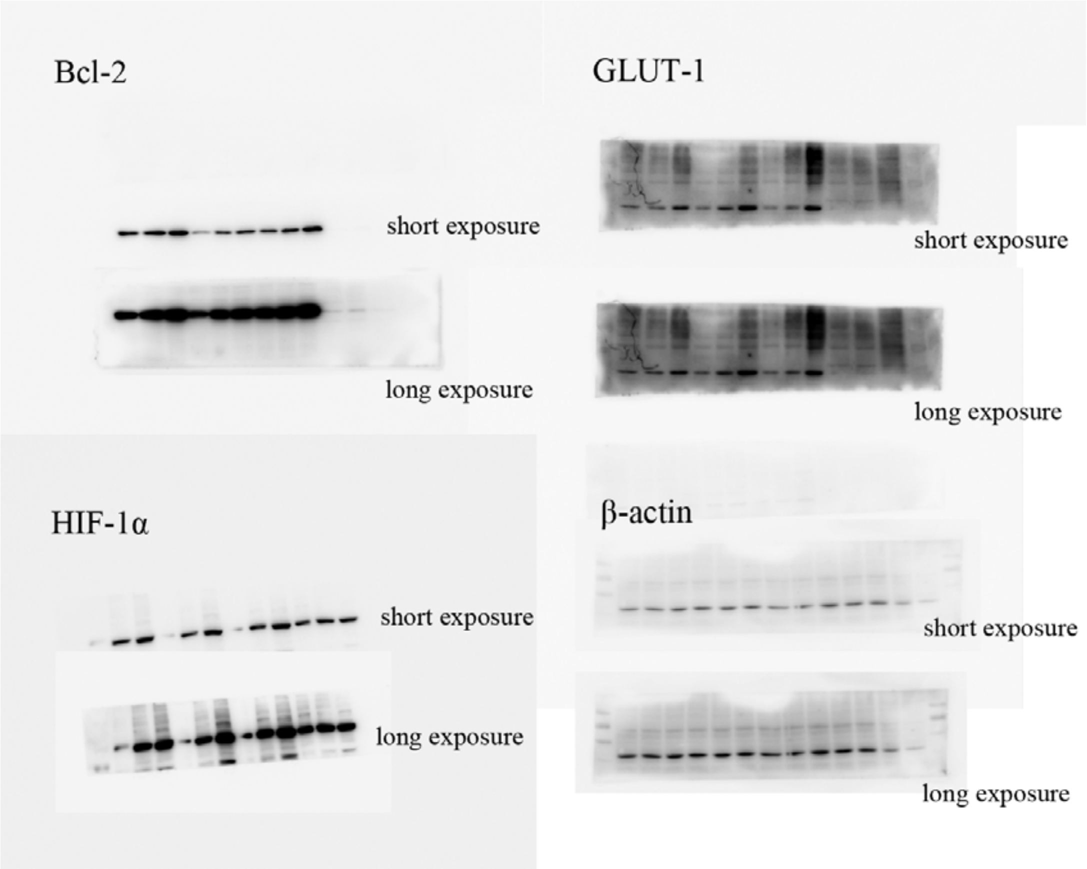

Supplementary Figure S3. Expression of GLUT-1, Bcl-2, and HIF-1α in HSC3, SCC3, TCA8113 and CAL27(The band of this cell line was not displayed in the cropped blots of the main paper) cells treated with or without metformin (10 μM) under hypoxic or normoxic conditions for 48 h.

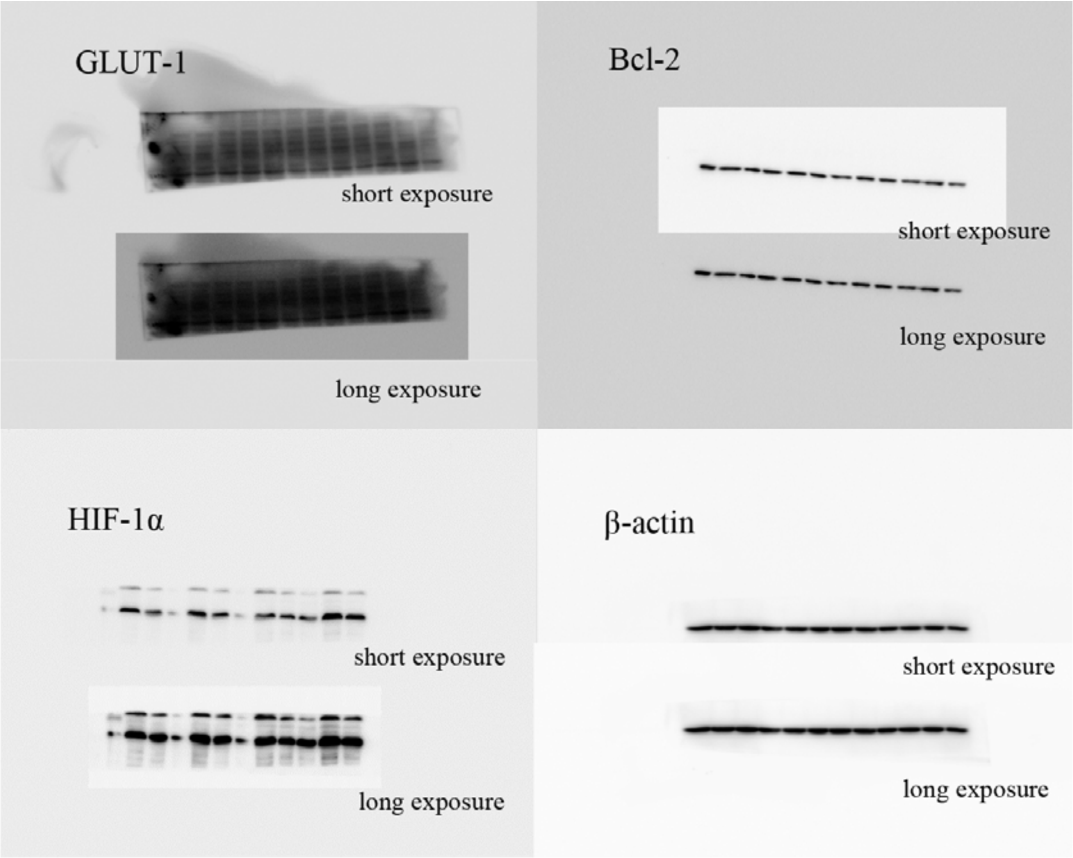

Supplementary Figure S4. Expression of HIF-1α, p65, and p-p65 in HSC3 cells transfected with or without p65 siRNA under hypoxic conditions for 48 h.

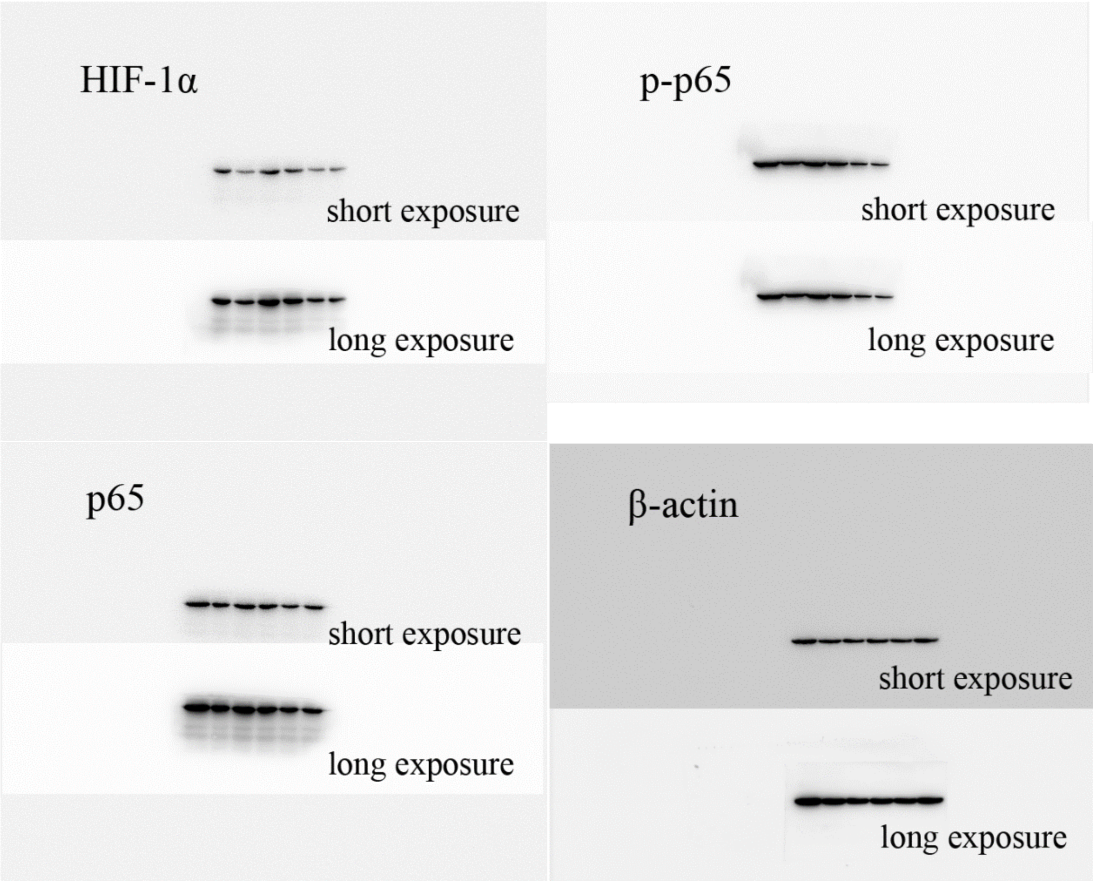

Supplementary Figure S5. Expression of p-AMPK and AMPK in HSC3, SCC3, and TCA8113 cells was detected under normoxic conditions, hypoxic conditions treated with or without metformin for 48 h.

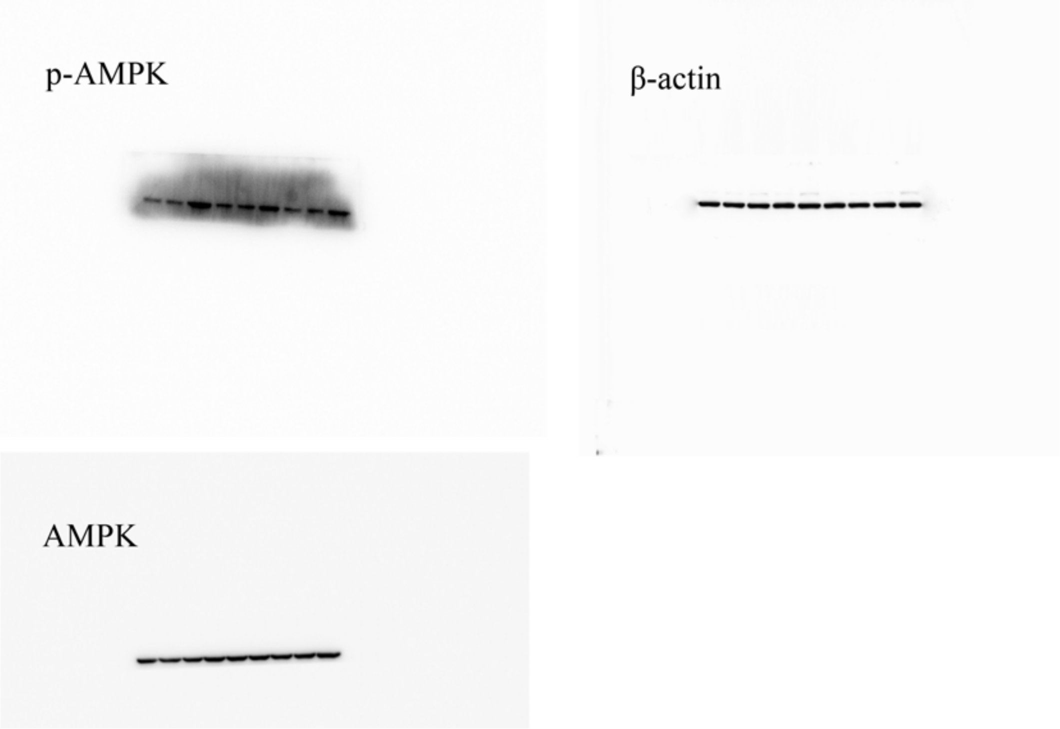

Supplement: Supplementary Information [file srep35788-s1.pdf]
